# Personality and predisposition to form habit behaviours during instrumental conditioning in horses (*Equus caballus*)

**DOI:** 10.1371/journal.pone.0171010

**Published:** 2017-02-03

**Authors:** Léa Lansade, Alain R. Marchand, Etienne Coutureau, Cyrielle Ballé, Floriane Polli, Ludovic Calandreau

**Affiliations:** 1 PRC, INRA, CNRS, IFCE, Université de Tours, Nouzilly, France; 2 Institut de Neurosciences Cognitives et Intégratives d’Aquitaine (INCIA), Université de Bordeaux, Talence, France; 3 Institut de Neurosciences Cognitives et Intégratives d’Aquitaine (INCIA), CNRS, UMR 5287, Talence, France; Universite de Lyon, FRANCE

## Abstract

The relationship between personality and learning abilities has become a growing field of interest. Studies have mainly focused on the relationship with performance, such as the speed of acquisition. In this study, we hypothesised that personality could in part also be related to a certain predisposition of an individual to switch more easily from a goal-directed process to a habit process during learning. To identify these processes, we conducted a contingency degradation protocol. This study investigated 1/ whether in general horses are able to adjust their response according to the contingency between their action and the reward, 2/ whether there are any relationships between certain personality profiles and a predisposition to switch more rapidly to habitual processes, and 3/ whether emotional states experienced during the learning procedure play a role in this switching. Personality tests were conducted on 29 horses, followed by a degradation contingency protocol. Overall, results show that horses were sensitive to contingency degradation between their action and the reward. Nevertheless, there was inter-individual variability: the horses presenting high fearfulness, and to a lesser extent low sensory sensitivity and low gregariousness were less sensitive to the degradation, demonstrating that they were more likely to switch to a habitual process. Contrary to our expectations, the emotional state experienced during the procedure did not seem to explain this switching. We conclude that personality is not only related to learning performance, but also in part to the process involved during learning, independently of the emotion experienced during the process. This study provides new theoretical knowledge on cognitive skills in ungulates.

## Introduction

Over recent decades, the inter-individual variability in the expression of behaviours and in cognitive abilities has become a growing field of interest in the scientific literature [1]. Along the same lines, relationships linking personality and certain learning abilities have been identified [2, 3]. For instance, recent research in a wide range of species has shown that the personality dimensions related to fearfulness or to exploration are linked to learning and memory performance, such as the speed of acquisition (ravens [4], guppies [5], horses [6–8], chickadees [9]). However, to date this research has mainly focused on the relationship with performance, i.e. how much or how fast a subject learns and remembers according to its personality. Yet, personality could also influence aspects other than simply learning performance. In fact, it is now accepted that for equal performance, different processes can play a role in learning. In the case of instrumental learning two processes have been described: goal-directed and habit-type processes [10–12]. A subject that has preferentially developed a goal-directed process is aware of the consequences of its actions, and thus of the contingency between the action and its outcome, and will act accordingly. Its behaviour will thus be more flexible. In contrast, a subject that has developed a habit-type process will form a link between its action and the preceding stimulus, rather than its consequences. This subject will thus have a more rigid automatic behaviour and become less sensitive to changes in the value of the outcome, and in the causal relationship between the action and its consequence.

Different factors can lead to a transition between the two processes. The first one is over-training. For instance, Tricomi et al. [13] have shown in humans that participants following an extensive training on a free instrumental task become less sensitive to outcome devaluation, suggesting a shift from a goal-directed to a habit process. An example of this over-training effect can also be illustrated in everyday life by a person learning to drive a car (see also [12] for more details). Beginners will begin by adopting goal-directed behaviour; they need to be very focused on the road but are flexible and for example, can easily drive on the right or the left side. With practice drivers will develop habits; their driving becomes more automatic and they are able to focus their attention elsewhere, but at the same time they are less flexible finding it more difficult to change sides. In addition to overtraining, a second factor could favour this switch during instrumental conditioning, namely stress [14]. For instance, students exposed to an acute stress before undertaking an instrumental task (hand immersed in iced water while being observed by an unfamiliar person) were more likely to develop habits rather than goal-directed actions [15]. Considering that sensitivity to stress is highly dependent on personality, we can postulate that certain personality profiles could predispose certain subjects to switch to habit processes. At least two experiments in horses support this hypothesis. First, using an instrumental procedure, Valenchon et al. [16] found that horses with a fearful personality were more resistant to extinction; this resistance may suggest the development of habitual processes. Second, we also found that fearful horses had a greater tendency to respond to Pavlovian instrumental transfer [17]. It has been reported that the development of habits favours this transfer [18]. This may therefore suggest that fearful horses are more likely to respond to the transfer because they have preferentially formed habitual responses. However, these results need further validation, using appropriate methodology. The aim of this study was therefore to determine formally whether there is a relationship between certain personality profiles and a predisposition to switch more easily to a habitual process and if this were the case, to determine whether this relationship was mediated by an emotional state (stress) experienced during the learning procedure.

To identify which processes are preferentially involved, a contingency degradation paradigm [19] was adapted for use with horses for the first time. Subjects were initially trained to perform a certain action to receive a certain reward. In the first ‘acquisition’ phase, the reward was given only after an action; there was a causal relationship between the action and the reward. In a second ‘degradation contingency’ phase, this causal relationship was degraded by increasing the probability that the reward would be provided independently of the action; the reward could be given either when the subjects performed the action or when they did not. Degrading the contingency in this way should have only a limited impact on the response rate of subjects which had developed habits as they would not aware of a causal link between their action and receiving a reward. In contrast, the response could be expected to decrease dramatically in subjects that had preferentially formed goal-directed responses. The horse is an interesting model in which to investigate this question because the concept of personality has been extensively studied in this species [20], including its relationship with learning abilities [6, 17, 21]. However, we do not know whether an ungulate such as the horse has the cognitive abilities required to detect different levels of contingency and to adjust its responses in consequence. By answering this question, we hope to improve our knowledge of cognitive skills in this species.

The present study aimed to investigate 1/ whether in general horses are able to adjust their responses according to the level of contingency between their action and the reward, 2/ whether there is a relationship between certain personality profiles and a predisposition to switch more rapidly to habitual processes, and also 3/ whether a specific emotional state experienced during the learning procedure plays a role in this switching. To this end, the personality of horses was assessed using a battery of behavioural tests aimed at measuring five dimensions: fearfulness, gregariousness, sensory sensitivity, activity level and reactivity to humans [22–25]. These dimensions have been shown to be stable across both situations and time; these forms of stability are fundamental to the modelling of personality dimensions [26, 27]. These dimensions are also independent from one another and thus measure different aspects of personality [25]. Finally, similar dimensions have been described in many other species, including humans [28]. This battery of tests has already been used several times to study the relationships between personality and learning abilities [7, 8, 16, 17, 21]. To determine whether habituation or goal-directed behaviours were preferentially involved, we then conducted a contingency degradation paradigm (for a detailed review on this paradigm, see [19]). The emotional state experienced by the animals during this paradigm was also evaluated through physiological (cortisol and heart rate) and behavioural measures.

## Materials and methods

### Animals

The study involved 29 non-pregnant mares (Welsh ponies, mean age 10.1 ± 1.7 years) bred at the French National Institute for Agricultural Research (INRA Nouzilly, France) and accustomed to being handled (regularly haltered and tethered). None of them had stereotypic behaviours. They had never been tested with this kind of procedure prior to the experiment.

Before the experiment, the animals lived in a group, at pasture in summer. In winter, they were housed in loose boxes (5 m × 3 m) in groups of three and spent 2 h per day together in a large outdoor paddock. The experiment was carried out under winter-housing conditions. The animals had straw bedding with water hay and mineral block available *ad libitum*. No additional food was given. During both personality and learning tests, the same unfamiliar non-participating ‘audience’ horse was tethered outside the test zone in order to be visible to the tested horse and to avoid a context of social isolation.

### Personality tests

On the first day, personality tests were conducted in a loose box (2.7 m × 8.1 m) to assess five independent dimensions (fearfulness, gregariousness, activity level, reactivity to humans and sensory sensitivity) as fully described in previous studies [22–25]. We used the behavioural parameters selected during these studies, as they appear to be reliable indicators of personality due to their stability over time (from a few months to a few years) and across situations. After a 5-minute period of habituation to the test pen, behavioural tests were carried out for a total period of approximately 30 min per horse. For more details, see Lansade and Simon [7]. Each horse followed the same test sequence:

- Human test (reactivity to humans): an experimenter unfamiliar to the horse entered the loose box and stayed motionless for 180 s. The number of contacts with the experimenter (sniffing or nibbling) was recorded. A high number of contacts with the experimenter indicated that the horse was “close to humans”.
- Tactile sensitivity test (sensory sensitivity): the horse was held by its halter, and an experimenter applied von Frey filaments (Stoelting, IL, USA) to the base of the horse’s withers. These filaments consist of a hard plastic body connected to a nylon thread calibrated to exert a specific force on the skin. To evaluate the reaction threshold of each horse, four different filament strengths were used, from the thinnest one (0.008g) to the thickest one (300g). The filament was applied perpendicularly on the animal’s skin until the nylon filament started to bend. We have previously shown that it is not possible to repeat the stimulation on a same area twice in a row, as generally the horse does not react the second time. For this reason, we changed sides between the first and the second filament, and we waited for several minutes between the first two and the last two filaments. So, in the first part of the test, we applied a 0.008 g filament to the right side of the horse and a 300 g filament to its left side. The same procedure was repeated several minutes later (after the novel area test), except that filaments of 0.02 g and 1 g were applied to the right and left sides, respectively. The response was coded in binary form (twitching/ not twitching) according to the reaction of the platysma muscle when the filament was applied and withdrawn. The number of times the horse reacted to the filaments being applied and withdrawn was recorded. This number ranged from 0 to 8. A high number of reactions indicated that the horse was more “sensitive”.
- Isolation test (gregariousness): The audience horse was removed so that the test horse could no longer, see, smell or hear it. The number of times the test horse neighed was recorded for 90 s. A high number of neighs indicated that the horse was more “gregarious”.
- Novel area test (fearfulness towards novelty): in order to eat from a familiar bucket containing pellets, the environment was arranged so that the test horse had first to cross a piece of colourful carpet. The latency to eat from the bucket was recorded. If the horse did not cross the area within 180 s, the test was terminated and a time of 181 s was assigned. The higher this latency was indicated horses were increasingly “fearful towards novelty”.
- Suddenness test (fearfulness towards suddenness): an umbrella was opened in front of the animal 3 s after it started eating from a bucket of pellets. The intensity of the startle response was recorded by measuring the flight distance (in metres). A high distance indicated that the horse was more “fearful towards suddenness”.
- Locomotor activity: the loose box was divided into six imaginary areas of equal size. The number of areas crossed during the habituation phase, human test, and isolation test were recorded. An increasing number of areas crossed indicated horses were increasingly “active”.

### Contigency degradation task

#### General principle

The aim of this task was to assess how sensitive the horses were to contingency degradation. To this end, the horses had first to learn to touch an object with their nose to obtain a reward. In this first phase, the contingency was positive; the reward was only given if they touched the object. During this phase the horses were trained with two objects at the same time, each associated with a specific food reward. In the second phase, the contingency was degraded for one of the objects. Thus, for the “degraded contingency” object, rewards were given both when the horse touched the object (contingent) and when it did not (non-contingent). For the “non-degraded contingency” object, the reward was only given after the horse touched the object (contingent). Finally, the horses were tested under an extinction condition–the objects were presented simultaneously and there was no longer a reward. An animal sensitive to contingency would be expected to touch the non-degraded object much more than the degraded object [29].

In order to avoid introducing bias in this experiment, and especially an effect of response competition, the protocol was made more complicated as suggested by different authors (review [19]). This “response competition effect” consists in the fact that under the degraded condition giving a non-contingent reward could encourage an alternative behaviour to appear, such as exploring the table on which the object was placed, thus entering into competition with the action of touching the object. In this case, if the animal decreased its response rate under the degraded condition, it would be impossible to determine whether this were due to response competition or sensitivity to contingency. Moreover, it was also important to distribute the same number of rewards under each condition (degraded or non-degraded). “Control” feed (unlinked to the object involved) was thus distributed non-contingently in the non-degraded condition. Under this condition two foods were therefore distributed: the reward food distributed contingently when the object was touched and the control feed given non-contingently.

#### Apparatus

**Fig 1:**
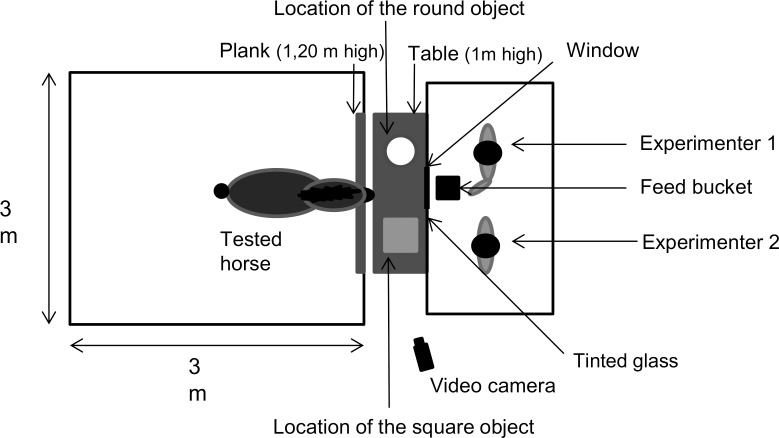
Schematic representation of the learning apparatus. During the “contingency degradation phase”, a zero contingency was associated with the square objet and the pellets, and a positive contingency was associated with the round object and the oats.

Horses were trained in a familiar loose box (3 m × 3 m) closed on three sides. The fourth side had an open window (1.20 m x 1.20 m) extending from the ceiling to 1.2m from the ground (Fig 1). A table (1 m high) was placed behind the window. On this table were fixed two plastic objects (0.60 m high) separated by 0.40 m: a round yellow and red object on the left (“the round object”) and a grey square object on the right (“the square object”). On the other side of the table, two observers were hidden behind a wall in which there was a small tinted window. A small trapdoor below the window allowed the observers to move a bucket of food onto the table.

#### Familiarization with the apparatus

Prior to learning, the horses were familiarized with the apparatus during 15-min sessions (2/day). The horse was led into the loose box, with both objects present on the table. During the first session, it was held in front of the objects with a 1.2-m leading rein each side and then for the subsequent sessions it was free. At 2, 5, 8 and 11 min after the beginning of the session, eight grams of alternatively pellets or oats were given in the bucket. The horses were familiarized with the apparatus for a minimum of three sessions or until they ate freely at each distribution of food (mean+-SE: 3.2 ± 0.8 sessions).

#### Acquisition of the instrumental task

Two 30 min sessions (one for each object), 5 hours apart (morning and afternoon), took place each day for 4 days (A1 to A4). During each session, only one object was present. The order in which the objects were presented was alternated every day. The aim was to train the animals to touch the object with their nose (nose-poke) to obtain a specific food reward (8 g of pellets for the square object and 8 g of oats for the round object). On the first day, horses were individually held in front of the objects with a 1.2-m leading rein each side and trained with a continuous schedule of reinforcement (every touch was rewarded). For the next 3 days, animals were free in the box and shifted to a variable interval 60-s schedule. This meant that nose pokes were not systematically rewarded, but that on average a nose poke was rewarded every 60 s. The rewards were given immediately after a nose poke. A reward was never given if the horse had not just touched the object.

#### Contingency degradation

Each day, two 30-min sessions (one for each object) took place 5 hours apart, for 6 days (D1 to D6). During each session, only one object was present. A positive contingency was associated with the round object and the oats. A zero contingency was associated with the square objet and the pellets. The association between a specific food and a specific level of contingency was randomly chosen. This took place after having conducted a preliminary experiment on 16 horses (different from those used in the final experiment), to check that 1/ there was no food preference for one kind of food, and 2/ there was no difference in terms of acquisition and degradation performances whether the non-contingent condition was associated with pellets (N = 8) or with oats (N = 8).

**Fig 2:**
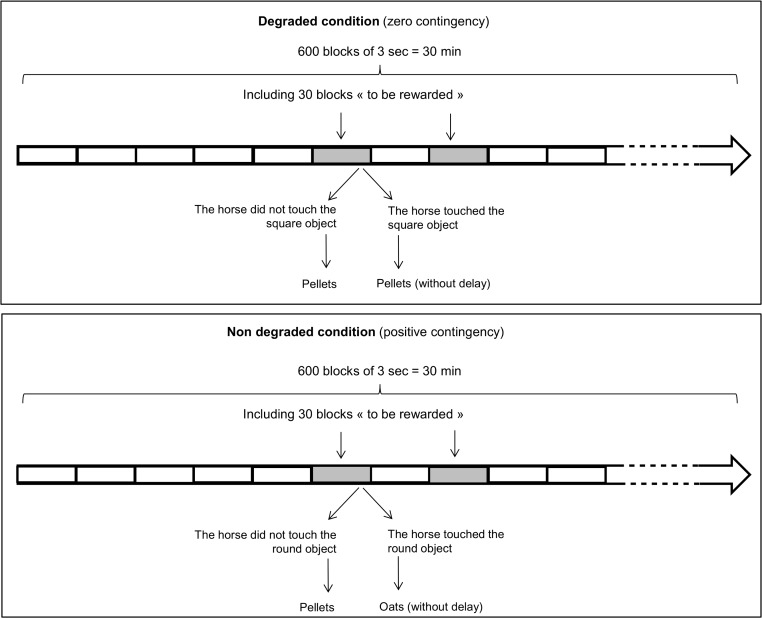
Overview of contingency degradation sessions. A positive contingency was associated with the round object and the oats. A zero contingency was associated with the square objet and the pellets.

During the final experiment, all the subjects underwent exactly the same procedure to avoid bias in the inter-individual comparisons. This choice implied not balancing the objects and the type of food between individuals. Each contingency degradation session was divided into 600 blocks of 3 s. (Fig 2). Thirty blocks were selected as "to be rewarded": a reward was systematically given during these blocks, but under two different conditions: degraded or non-degraded. These blocks were randomly distributed over the session.

Under the degraded condition (zero contingency) the pellets were given independently of the nose-poke. Thus, during a “to be rewarded” block, the horse received pellets whether or not it had touched the square object with its nose (i.e. the horse could get pellets “for free”).

Under the non-degraded condition (positive contingency) the oats were always given contingently with a nose-poke (i.e. the horse could never get oats “for free”). Thus, during a “to be rewarded” block, if the horse had touched the round object with its nose, it immediately received the oats. If it had not, it did not receive the oats, but it received pellets. As we explained above, this procedure was selected to control for the effect of response competition and to equate food-intake time under the two conditions while maintaining for the non-degraded object a schedule as similar as possible to that for acquisition.

#### Choice extinction test

To evaluate the effects of contingency degradation training, the horses received a 30 min choice extinction test with both objects present and no reward distributed.

### Behavioural and physiological parameters measured during acquisition and degradation sessions

#### Indicators of cognitive performance

The number of nose-pokes on each object was recorded during each session of acquisition and degradation, called here the “nose poke rate”. In degradation sessions and in the choice extinction test, a “relative nose poke rate” was calculated, corresponding to the ratio between the number of nose pokes during degradation or extinction sessions and the baseline rate during the last acquisition session (A4). A high relative nose poke rate for the degraded object (square object) was considered to indicate performance under habitual processes.

#### Indicators of stress

During each acquisition and degradation session, the number of snorts (forceful expulsion of air from the nostrils) was recorded, as a behaviour indicating stress experienced during the learning task. Average number of snorts per session were calculated for each phase (acquisition or degradation). Neighs as an indicator of social stress would also have been recorded, but this behaviour was never expressed. Two physiological stress indicators were also measured: cortisol concentration and heart rate. To assess salivary cortisol concentration, saliva samples were collected with Salivette® Cortisol (Sarstedt France). Samples were collected just before and just after the first session of acquisition, and the first session of degradation. The cotton buds used for sampling were centrifuged at 4000 g for 10 min at 4°C, and the saliva was stored at -20°C until analysis. Cortisol was measured using a luminescence immunoassay kit (LIA, IBL, Hamburg, Germany). The measurements were performed in replicate. The intra-assay coefficients of variation were 8.6% and 11.3% at 1.8 ng/ml and 8.67 ng/ml, respectively. The assay sensitivity was 0.3 ng/ml. In the analyses, we considered the ratio between the concentration measured just after a session of acquisition or degradation and the concentration measured just before, called here the “cortisol ratio”. The heart rate was recorded during the same sessions as for the cortisol measurements. The heart monitor system (Polar Equine RS800CX Science, Polar Oy, Finland) consisted of a flexible belt with two integrated electrodes, a transmitter, a separate storage device, and the corresponding software (Polar Pro Trainer, Version 5). The electrodes were placed behind the left humerus and across the sternum and electrical conductivity was enhanced using echographic gel. The storage device was fixed to the belt within range of the transmitter. Animals were habituated to the polar device during the first three sessions of familiarization to the apparatus. The mean HR in beats/min was calculated for each whole session, called here “heart rate”.

### Statistical analyses

Statistical tests were performed with Xlstat 2013 software (Addinsoft Inc., France). Parametric analyses were conducted on “nose-poke rates” and “relative nose poke rate”. The changes in “nose-poke rates” over the four acquisition days (A1 to A4) were analysed using Anova. Two-tailed paired samples t-tests were used to compare the “nose poke rates” in the degradation days (D1 to D6) with the baseline (A4). For the choice extinction test, the “relative nose poke rate” was compared between the two objects with a two-tailed paired samples t-test. The results of each acquisition and degradation session were expressed as mean +SE. The level of statistical significance was set at *P* < 0.05. Multiple Factor Analysis (MFA) was used to determine the relationships between the personality profiles and the nose pokes during acquisition or degradation. The advantage of this analysis is that it identifies which combination of dimensions (i.e. a personality profile) could be related to cognitive performance, and not only the relation with each dimensions individually. This analysis included the six variables measured during the personality tests for the first set of data. The “nose-poke rate in acquisition” and the *“*relative nose poke rate in degradation” were used for the other data sets. The “nose-poke rate in acquisition” was the number of nose-pokes on the square object during the last acquisition session (A4) and was considered as an indicator of performance during acquisition. The “relative nose poke rate in degradation” was the relative nose-poke rate for the last session (D6) under the degraded condition (square object). It was considered as our indicator of a habitual process. The analyses focused only on the square object as this enabled us to compare firstly the relationship between personality and nose pokes during acquisition (first phase), and secondly between personality and nose pokes during degradation (second phase). This analysis was based on non-parametric tests because of the lack of normality in the personality data. The percentage of variability explained by the first four factors, and the Spearman correlation coefficients calculated between these factors and the variables are presented in the results. Finally, Spearman correlation coefficients were calculated between the “nose-poke rate in acquisition” or “relative nose-poke rate in degradation” and the three indicators of stress measured during the corresponding phase (snorts, cortisol ratio and heart rate).

### Compliance with ethical standards

The protocol was approved by the ethics committee of Val de Loire (N°2012-12-23 delivered by CEEA Val de Loire). Animal care and experimental treatments complied with the guidelines of the French and European guidelines for the accommodation and care of animals used for scientific purposes (European Union Directive 2010/63/EU) and were performed under authorization and supervision of official veterinary services (agreement number C-37-125 delivered to the UEPAO animal facility by the veterinary service of the Département d’Indre et Loire, France). At the end of the experiment, the animals returned to normal rearing conditions. No signs of injury or pain were observed during or after the experiments. No stress was induced during the experiments. The horses lived in social groups and were taken to an outside paddock daily. The horses were not food restricted during the entire experimental period.

## Results

### Nose poke rate during acquisition, degradation and extinction

**Fig 3:**
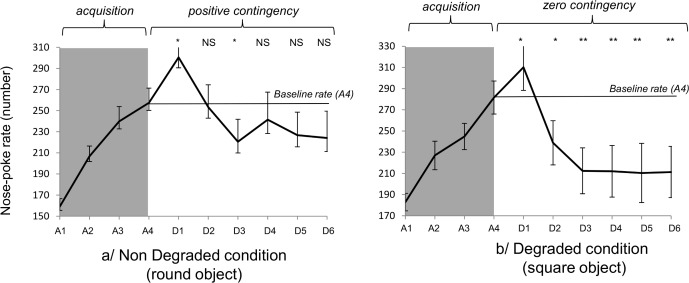
Nose-poke rate during acquisition and degradation sessions under the a/non-degraded condition and b/ degraded condition. The statistics correspond to the comparison between the number of nose pokes during the degradation sessions and the baseline rate (t test). The baseline rate is the number of nose pokes performed in A4. NS: Non-significant *: P<0.05 **: P<0.01 A1 to A4: first to fourth day of acquisition D1 to D6: first to sixth day of degradation Non-degraded condition: round object Degraded condition: square object.

**Fig 4:**
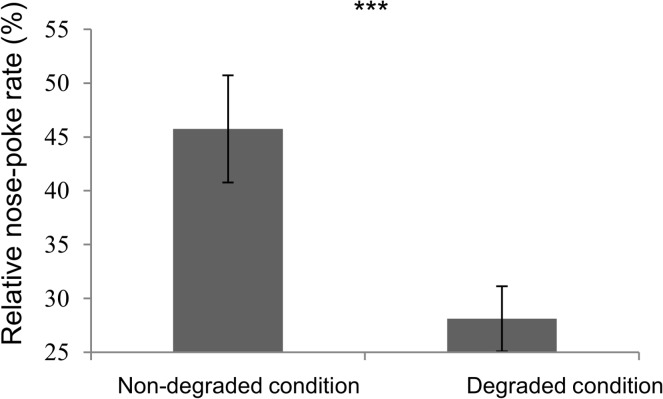
relative nose poke rates in the non-degraded and degraded conditions during the choice extinction test. Relative nose poke rate: number of nose-pokes in extinction / baseline rate in A4 ***: P<0.001 *t* test.

The horses gradually increased nose-poke rates over the four acquisition sessions (A1 to A4), for both the round and the square objects (*F*3,84 = 35.86, *P*<0.0001; *F*3,84 = 32.15, *P*<0.0001 respectively, Fig 3A and 3B). When the condition was degraded for the square object (Fig 3B), the nose-poke rate first increased significantly at D1, and then became statistically lower than the baseline rate from sessions D2 to D6. By contrast, in the non-degraded condition (Fig 3A), after a significant increase at D1, the nose-poke rate did not differ significantly from the baseline rate, except in session D3. Finally, during the choice extinction test (Fig 4), the relative nose-poke rate (number of nose-pokes in extinction / baseline rate in A4) was significantly lower in the degraded than in the non-degraded condition (*t28* = 4.35, *P*<0.001).

### Relationships between nose-pokes and personality profile

**Fig 5:**
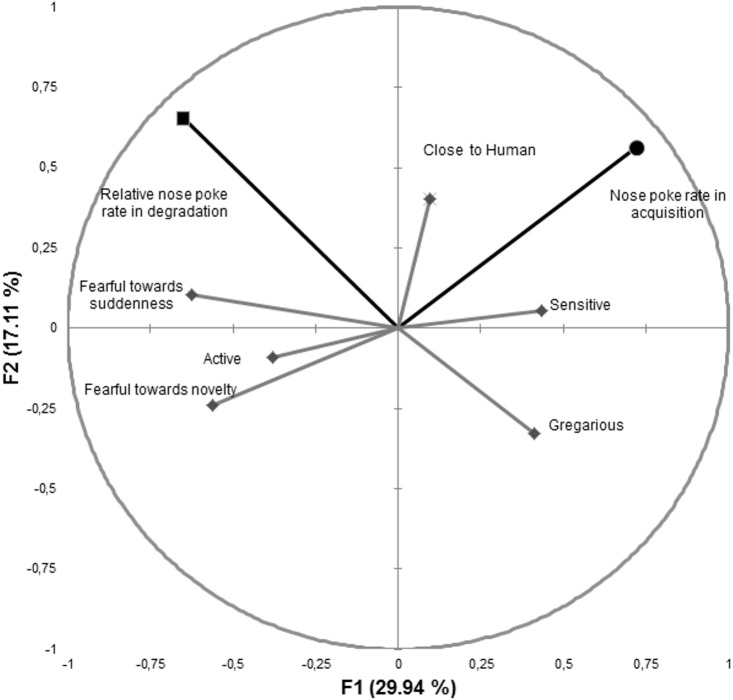
Correlation circle corresponding to the multiple factor analysis including personality data and nose-poke rate. ◆Personality data ● Nose poke rate in acquisition ■ Relative nose-poke rate in degradation.

The first four factors explained 71.24% of the total variance (Fig 5 and Table 1). The variables “nose-poke rate in acquisition”, “relative nose poke rate in degradation”, “fearful towards suddenness” and “fearful towards novelty” were mainly correlated with F1. “Active” and “close to humans” were mainly correlated with F3. “Sensitive” and “gregarious” were mainly correlated with F4.

**Table 1:** Multiple factor analysis including personality data and nose-poke rate.

	F1	F2	F3	F4
	*29*.*94%*	*17*.*11%*	*12*.*57%*	*11*.*62%*
Nose poke rate in acquisition	**0.72**	0.56	-0.15	0.07
Relative nose poke rate in degradation	**-0.66**	0.65	-0.04	0.11
Fearful towards suddenness	**-0.62**	0.11	-0.02	-0.08
Fearful towards novelty	**-0.56**	-0.24	-0.19	0.17
Sensitive	0.43	0.06	0.29	**-0.67**
Gregarious	0.41	-0.33	0.23	**0.69**
Active	-0.38	-0.09	**0.71**	-0.15
Close to Humans	0.10	0.40	**0.67**	0.29

The table shows the percentage of variability (*italic*) explained by the first four factors (F1 to F4) and the Spearman correlation coefficients (r value) between the factors and the variables. Values in bold indicate for each variable the factor for which the correlation coefficient is the highest.

Since we were particularly interested in the learning variables, and because they were the most strongly correlated with F1, we focused essentially on this factor. Interestingly, “nose-poke rate in acquisition” was positively correlated with F1, whereas “relative nose-poke rate in degradation” was negatively correlated with it. As a whole, this analysis suggests that the horses with a personality profile showing little fearfulness were more likely to touch the object during acquisition. By contrast, during contingency degradation, the horses presenting high fearfulness had a greater tendency to display a high relative nose poke rate during contingency degradation, thus suggesting a habitual response. In addition to a clear relationship with fearfulness, coefficient correlation with F1 also suggests a certain relationship with sensory sensitivity (R = 0.43) and even gregariousness (R = 0.41).

### Relationships between nose-poke rates and indicators of stress

The “nose poke rate in acquisition” and “relative nose poke rate in degradation” were never significantly correlated with the three stress indicators: number of snorts, cortisol ratio, and heart rate measured during the corresponding sessions (Table 2).

**Table 2:** Relationships between nose-poke rates and indicators of stress.

**Acquisition**		Correlations with nose poke rate in acquisition
Heart Rate (bpm)	46.5 (44.7; 54.6)	R = -0.01; P = 0.98 NS
Cortisol ratio	0.52 (0.42; 0.94)	R = 0.11; P = 0.55 NS
Snorts (average number)	0.25 (0; 0.5)	R = -0.14; P = 0.47 NS
**Degradation**		Correlations with relative nose poke rate in degradation
Heart Rate (bpm)	45.6 (40.71; 50.08)	R = -0.25; P = 0.20 NS
Cortisol ratio	0.55 (0.47; 0.67)	R = -0.12; P = 0.54 NS
Snorts (average number)	0.17 (0; 0.5)	R = -0.23; P = 0.23 NS

Median and interquartile range (IQ1;IQ3) of the indicators of stress and Spearman correlation coefficient calculated between these indicators and the nose poke rate in acquisition, or the relative nose poke rate in degradation (R and P values are presented). bpm: beats per minute; NS: Non Significant

## Discussion

Our results show that overall horses were sensitive to contingency degradation between their action and the reward; they gradually decreased the number of times they touched the square object when the contingency was degraded. In addition, during the extinction test they touched the degraded object fewer times than the non-degraded object. Nevertheless, there was an inter-individual variability in the nose poke rate, both during the acquisition and contingency degradation phases. This variability was linked to specific personality profiles. Horses with a personality profile showing high fearfulness and to a lesser extent low sensory sensitivity and low gregariousness tended to perform worst during acquisition (they touched the object the less), but on the other hand, they were more likely to display a higher relative nose poke rate during contingency degradation, suggesting a predisposition to develop habitual responses more easily.

### The nose poke rate was associated with specific personality profiles according to the phase

A positive relationship between acquisition performance and a personality profile showing little fearfulness (or related dimensions such as stress vulnerability or anxiety) and/or high sensory sensitivity has previously been reported in different species (horse [7, 17], rodent [30]). Our study further supports this relationship. However, this is the first time that a link between a specific personality profile and a predisposition to form habits has been demonstrated through an appropriate experimental procedure. Previous studies that had suggested such a link were based on extinction processes, which were more difficult to interpret. Those previous studies showed that individuals from stress sensitive lines or having a fearful personality were more resistant to extinction (horse [16], rodent [31]). Valenchon et al. [16] also described that horses presenting a low level of sensory sensitivity were more resistant to extinction. This resistance to extinction could reveal a predisposition to develop habitual processes [32], but it could also be interpreted in different ways, such as a change in cognitive flexibility [33] or demonstrating exploratory behaviour [34]. In contrast, our contingency degradation protocol enabled a more specific conclusion to be drawn in terms of which process was preferentially developed.

The interesting aspect of the present results is that the personality profiles associated with the nose poke rate differed according to the phase. In particular, the fact that fearfulness was negatively linked to the nose poke rate during acquisition but was positively related to it during degradation clearly shows that the influence of fearfulness is specific to the cognitive process involved, and does not only influence the behavioural response (i.e. the motor response of touching the object with the nose). Indeed, if the fearfulness dimension had only influenced the behavioural response of touching the objects simply through the animals’ fear of them, the relationship would have been the same in the two phases.

Having established this relationship between personality and cognitive ability, the question is whether this link could be mediated by the emotional state (stress) experienced during the tests. This is a postulate suggested in a recent paper by Griffin et al. [1]. Indeed, it could be thought that through a cascade effect personality dimensions would induce specific emotional states during learning procedures and these in turn could affect cognitive performance. In the present study this was a strong assumption, notably regarding fearfulness: a fearful personality could have induced stress during the procedure, stress which is recognized for decreasing acquisition performance [21] and promoting a switch to a habit process [14]. Yet, contrary to our expectations, the three indicators of emotional state (heart rate, cortisol ratio and snorts) measured during the sessions were not correlated with the nose poke rate, whatever the phase. It is probable that the learning process did not induce stress even in the most fearful horses. Thus, the switch to a habit-type process could not be explained by stress. Furthermore, no neighing (behaviour indicating social stress) was recorded during the procedure, again showing that the gregarious profile did not induce a particular emotional state which could influence cognitive skills. Other explanations therefore need to be found to understand why certain personality profiles would be associated with certain cognitive abilities. One such explanation could be related to an evolutionary process. There could have been co-selection of certain personality profiles and cognitive abilities over time which could have conferred an adaptive advantage on individuals. For example, fearfulness when associated with a capacity to switch easily to a habitual system, could have procured an adaptive advantage enabling fearful animals to maintain their on-going activity under the form of a habit-type behaviour, while at the same time looking out for potential dangers. Indeed, the benefit of such a dual system is multitasking. The goal-directed system can be involved in the most difficult tasks, while the habit system executes background tasks [35].

In the case of sensory sensitivity, the relationship is more tenuous than for fearfulness and needs to be confirmed. However, several studies have already reported relationships between individual differences in sensory-processing sensitivity and variation in different types of behavioural plasticity, including flexibility [36]. In the present case, we can suspect that the most sensitive subjects would not only be the most aware of sensorial changes in their environment but also to cognitive-type changes. This awareness would render them more able to adapt and more flexible, a feature of a goal-directed response. Nevertheless, this remains hypotheses and much more research is required to improve understanding of the mechanisms underlying the relationships between personality and cognitive performance. Finally, the link with gregariousness, which has not been described in the literature previously, needs to be confirmed before discussing it further.

### These results improve understanding of the cognitive capacities of horses

The fact that overall, horses were able to adjust their response according to the contingency between their action and the reward is in itself significant because it provides new knowledge on the cognitive skills of an ungulate such as the horse. Indeed, the mental process underlying this ability is thought to be relatively sophisticated. Dolan and Dayan [37] suggested that the process required prospective planning involving a decision tree of future states and possible actions constructed from a model of the environment which had to be learnt. In addition, it is important to remember that our experimental procedure was particularly complex to avoid different interpretation biases and involved giving a non-contingent reward, whatever the condition. Only the type of reward changed between the two conditions. The fact that the horses had different response rates according to the conditions shows that they were able to create a precise mental representation of the consequences of their actions and of which reward was associated with each action. This also demonstrates that they were able to adapt their response level to obtain the reward they wanted. Moreover, certain authors have also drawn comparisons between goal-directed/habit-based systems and conscious/unconscious systems [35, 38]. In this way, our study highlights experimentally the existence of complex cognitive processes in ungulates such as horses, which probably involve the subject forming a conscious mental representation of the consequence of its actions, including the causal status of these actions.

These results are also of interest to optimize and tailor training and living conditions of horses. In particular, a common practice in the field consists in giving the same kind of food to reward the animal during training, but also at any other moment, for instance to improve the relationship with the animal. The fact that the horse is sensitive to the contingency between its action and the associated reward suggests that this practice is inappropriate and tends to demonstrate how important it is to use a specific kind of food reward for a desired action and never to give this food under other circumstances. This is especially true for unfearful horses. Moreover, the predisposition of the most fearful horses to switch more easily to a habit-type response certainly has an incidence on the capacity of domestic horses to adapt to their conditions of training and captivity. On the one hand, this predisposition may be an asset for training since riders seek horses that respond to their aids more rapidly and automatically, and a horse that has developed habits may do this independently of reinforcement. This may contribute to explaining why fearful horses are popular with experienced riders despite being more complicated to ride [39, 40]. On the other hand, this propensity for habit processes may represent a risk factor for the most fearful subjects to develop psychopathologies, in particular stereotypies of animals kept in captivity, such as horses [41, 42].

In conclusion, this study provides proof that personality is not only linked to how much and how fast an individual can learn but also to the way it learns depending on the specific contributions of different systems: habit vs goal-directed. Furthermore, it provides new theoretical knowledge on cognitive skills in equine species, suggesting that ungulates such as horses can form a mental representation of the consequences of their actions.

## Supporting information

S1 File Data set. (PDF)
