# Supplementary material for: Personality and predisposition to form habit behaviours during instrumental conditioning in horses (Equus caballus)
Source: PLoS One. 2017 Feb 3;12(2):e0171010. doi: 10.1371/journal.pone.0171010 (PMC5291538; doi:10.1371/journal.pone.0171010)
Supplement: S1 File — (PDF) [file pone.0171010.s001.pdf]

animal number	Human test	Sensitivity test	Novel area test	Suddenness test	Activity	Isolation test
**1**	0	6	17	1,25	13	3
**2**	17	4	10	2,75	145	5
**3**	13	0	10	4	62	0
**4**	0	2	181	3,5	79	5
**5**	6	3	8	2,5	137	1
**6**	15	8	13	3	127	3
**7**	3	5	11	2,75	119	3
**8**	8	6	7	1,75	87	2
**9**	13	8	6	2,5	89	3
**10**	8	8	10	1,75	45	3
**11**	5	8	181	3,25	139	0
**12**	0	3	181	1,5	93	4
**13**	5	7	7	1,75	43	5
**14**	0	8	17	2,75	77	1
**15**	14	7	10	2,25	61	2
**16**	6	3	181	3,5	61	2
**17**	7	6	9	1,5	76	6
**18**	0	1	39	3,25	40	0
**19**	3	6	8	3,5	48	5
**20**	18	4	42	1	42	5
**21**	13	4	38	2,5	48	4
**22**	3	2	5	3,25	116	5
**23**	9	7	48	1,75	70	2
**24**	0	8	7	3,25	58	1
**25**	0	8	23	2	83	0
**26**	0	7	6	1	92	6
**27**	0	5	6	2	79	2
**28**	0	2	12	2,25	66	4
**29**	0	4	10	1,85	79	3

nose pokes A1 square	nose pokes A2 square	nose pokes A3 square	nose pokes A4 square	nose pokes D1 square	nose pokes D2 square	nose pokes D3 square
179	161	192	277	230	256	69
170	219	277	357	456	292	305
209	203	203	269	401	319	339
132	160	177	233	167	170	152
189	284	222	295	251	240	185
150	150	187	230	194	85	93
89	193	221	220	222	107	70
226	340	321	412	498	457	380
198	209	218	244	222	111	91
160	369	327	437	595	406	207
133	187	167	159	108	290	437
173	157	176	190	290	158	101
229	382	403	470	543	212	268
191	269	270	274	394	165	223
158	149	184	206	228	141	130
200	180	221	216	213	177	216
190	262	292	387	312	131	115
208	235	269	300	341	339	304
205	331	304	333	241	238	302
236	188	234	276	318	359	191
59	145	176	212	261	283	206
183	166	173	199	236	220	194
199	350	393	454	452	529	568
279	303	314	294	441	216	221
219	250	265	279	302	133	100
203	207	187	208	267	124	115
151	181	227	184	246	126	147
160	177	189	273	260	353	212
226	176	310	277	304	291	219

nose pokes D4 square	nose pokes D5 square	nose pokes D6 square	nose pokes A1 round	nose pokes A2 round	nose pokes A3 round	nose pokes A4 round
106	115	94	208	310	204	264
517	542	425	90	191	266	264
342	348	372	160	188	239	323
99	40	93	125	118	182	198
100	57	47	151	166	269	247
139	146	180	151	143	145	140
65	71	66	110	192	175	211
314	299	315	155	250	301	359
85	79	13	150	223	228	235
330	308	245	190	266	381	311
313	338	240	136	206	168	203
141	110	153	198	225	156	192
265	280	132	255	331	406	436
132	176	302	174	222	255	263
113	134	136	106	167	125	155
143	110	202	146	196	195	215
48	70	52	143	249	253	289
334	425	368	133	216	298	302
225	179	157	167	179	281	331
363	388	268	170	180	242	234
237	251	176	83	101	161	184
234	162	256	127	140	176	170
567	648	628	243	233	410	458
148	96	234	164	279	321	275
153	167	223	172	175	247	237
95	77	209	215	198	152	204
144	139	88	158	184	210	205
261	206	243	181	204	290	283
135	140	210	153	263	218	275

nose pokes D1 round	nose pokes D2 round	nose pokes D3 round	nose pokes D4 round	nose pokes D5 round	nose pokes D6 round	relat. nose poke rate extinction square
341	234	263	316	303	302	17,69
362	202	235	320	400	383	29,69
485	408	385	409	410	450	77,32
213	212	217	132	231	216	13,73
315	301	170	152	163	67	17,97
144	48	29	9	15	9	21,30
178	262	150	136	111	142	7,73
415	383	276	332	321	171	20,39
234	172	112	181	121	20	13,93
361	351	221	289	244	301	14,87
224	305	188	317	235	201	54,72
174	275	138	107	234	180	62,63
342	127	120	187	61	48	21,91
250	107	152	133	156	176	24,82
183	158	103	79	81	96	25,73
246	236	145	154	178	111	15,28
337	169	233	176	160	197	28,94
380	383	355	366	384	489	22,67
343	412	369	366	260	279	19,52
336	228	307	422	256	278	41,67
188	227	157	175	207	94	17,92
177	192	167	177	155	126	24,62
634	549	585	704	545	576	35,24
396	152	177	303	266	369	37,41
302	121	101	141	122	205	21,51
262	210	210	150	166	237	41,35
281	283	291	238	234	250	21,74
364	430	382	392	378	303	12,82
248	212	158	137	177	221	50,18

relat. nose poke rate extinction round	heart rate acquisition	cortisol ratio acquisition	snorts acquisition	heart rate degradation	cortisol ratio degradation	snorts degradation
57,95	61,17	0,70	4,00	47,81	0,67	12,00
81,06	45,52	0,75	0,25	40,71	0,57	0,17
70,90	48,77	0,52	0,25	45,49	0,82	0,50
71,72	41,40	0,45	4,25	38,82	0,53	2,33
21,05	45,53	0,40	0,00	54,49	1,12	0,67
7,86	41,03	0,08	0,00	39,97	0,66	0,00
17,54	45,23	0,56	0,00	43,38	0,75	0,17
16,99	61,81	1,12	0,25	47,81	0,15	0,33
8,94	66,34	1,11	0,00	43,84	0,79	0,00
72,99	44,72	0,94	0,25	41,31	0,57	0,33
69,46	42,92	0,05	2,50	40,71	0,00	0,17
103,65	54,58	0,38	0,25	56,17	0,50	0,00
22,02	64,33	0,01	0,00	69,76	0,50	0,00
24,33	50,34	0,94	2,00	37,48	0,24	0,17
18,06	64,98	0,56	0,50	79,92	0,19	0,17
13,02	53,14	0,45	0,00	37,23	0,66	0,00
53,63	35,14	0,40	0,00	35,21	0,41	2,00
27,48	53,81	1,13	2,75	50,08	1,46	2,83
34,44	63,37	0,48	0,25	49,71	0,68	0,50
64,96	52,09	0,50	0,25	44,76	0,61	0,17
29,35	44,81	0,60	0,00	54,38	0,24	0,00
29,41	36,33	0,49	0,00	35,00	0,67	0,00
42,36	39,32	0,62	0,00	38,07	0,47	0,00
60,00	46,51	0,25	1,00	51,52	0,54	2,17
46,41	33,11	0,45	0,00	72,53	0,37	0,17
94,12	46,06	0,42	0,25	45,60	0,55	0,00
35,61	59,38	1,55	0,75	47,59	0,48	0,17
68,55	50,58	1,37	0,25	48,31	0,75	0,17
62,55	46,51	0,94	0,00	48,91	0,55	0,00
